# Knowledge and Attitudes Regarding Do-Not-Resuscitate Decisions Among Clinical-Year Medical Students and Interns in the Western Region of Saudi Arabia

**DOI:** 10.7759/cureus.52558

**Published:** 2024-01-19

**Authors:** Hasan Z Binjabi, Mohammed H Alturki, Abdulaziz A Almtrfi, Abdullah A Qashar, Abdullah M Alobedi, Hazem M Aljabri, Abdulaziz O Babukur, Muhanna M Almatrafi, Rayan O Almalki, Mohammad S Dairi

**Affiliations:** 1 Department of Medicine and Surgery, College of Medicine, Umm Al-Qura University, Makkah, SAU; 2 Department of Medicine, College of Medicine, Umm Al-Qura University, Makkah, SAU; 3 Pulmonary/Critical Care Medicine & Cardiac Surgery Intensive Care Unit, King Faisal Specialist Hospital & Research Centre, Jeddah, SAU

**Keywords:** do not resuscitate, awarness, cardiopulmonary resuscitation, saudi arabia, western region, attitude, knowledge, dnr, critical care

## Abstract

Background

The directive to withhold emergent interventions in the case of cardiac and/or respiratory arrest with the continuation of standard care and therapy is known as do-not-resuscitate (DNR). The diversity of DNR guidelines depends on moral and religious factors. In Saudi Arabia, a DNR policy was published in 2017 which corresponds to the religious and ethical aspects of Islamic law. To augment future awareness regarding DNR decisions, as they are an essential element in critical care medicine, the foundational principles of DNR must be provided during the clinical years of medical school.

Objectives

The current study aims to assess and evaluate the knowledge and attitudes regarding DNR decisions among clinical-year medical students and interns in the Western Region of Saudi Arabia.

Methods

A cross-sectional study was carried out from October to December 2023, utilizing a self-administered online survey distributed via social media platforms. After receiving ethical approval from the institutional review board, data were collected from clinical-year medical students and interns in the Western Region of Saudi Arabia, and an appropriate statistical analysis was performed.

Results

A total of 397 participants were enrolled in this study. More than half (n = 251, 63.2%) were from Umm Al-Qura University, while the remaining (n=79, 19.9%) were from Taibah University. Of the total, 258 (65%) were male participants, and 139 (35%) were female. A total of 152 (38.3%) were fifth-year medical students, and 102 (25.7%) were interns. The vast majority (n = 364, 91.7%) had heard the term DNR, with the most reported source of information being from healthcare providers (n = 306, 83.2%), while a minority (n = 33, 8.3%) had not. Of the respondents, 226 (56.9%) identified the presence of a clear DNR policy in Saudi Arabia, and 77 (19.4%) had previously had experience with DNR. Most of the studied population (n = 333, 83.9%) expressed a willingness to take a lecture/session regarding DNR. Most of our participants, 347 (87.4%), believe it is essential to consider legal concerns when making a DNR decision. Interestingly, 152 (38.5%) of the participants think it is acceptable to be conservative in investigations and treatments with patients who are labeled as DNR, and 223 (56.2%) agree that patients should be aware of their DNR status. Approximately three-quarters of the study population (n = 290, 73%) agreed that it is stressful to discuss the possibility of a DNR order. In the association of who heard about DNR more, 101 (99%) of the interns had heard about the term DNR, while only 53 (75.7%) of the fourth-year medical students had. At the same time, 74 (72.5%) of the interns showed a positive attitude regarding the DNR definition, compared to 33 (47.1%) of the fourth-year medical students.

Conclusion

This study highlights the necessity of integrating educational interventions into DNR decisions in addition to clinical placement in the intensive care unit as part of the medical school curriculum.

## Introduction

In 2004, the National Institutes of Health defined end-of-life care as the care provided to a person during the final stages of life. However, there is no specific definition of life’s final stages, nor can it be precisely foreseen [1].

Cardiopulmonary resuscitation (CPR) was first introduced by Kouwenhoven in 1960 and was initially known as closed chest cardiac massage [2]. CPR is an emergency intervention performed on an individual in cardiac and/or respiratory arrest [3]. Consequently, it was recognized that CPR inflicted suffering on certain patient groups instead of adding any significant benefit, which led to the emergence of do-not-resuscitate (DNR) orders in clinical practice [4].

A DNR order is a clinical order consented to by the patient or a proxy to withhold CPR during cardiac or pulmonary arrest without halting standard care and therapy [5].

DNR policies and guidelines are diverse since making such a decision depends on moral and religious factors. In Saudi Arabia, the Saudi Health Council published the DNR policy in 2017 on the basis of a Fatwa (a legal opinion on the point of Islamic law) in 1988 No. 12086. The DNR policy stipulates that a DNR order is established by three physicians, including the primary consultant, for cases in which resuscitation is futile. Family members are not permitted to intervene in the decision; however, they must be duly informed. The order should specify which procedures are included: intubation and ventilation, chest compressions, or ionotropic support. Nevertheless, all other treatment modalities, such as comfort and support care, may still be administered [6].

Previous studies have been conducted among medical students and interns in Jeddah, Saudi Arabia, Hong Kong, and Iran [7-9]. However, no study has yet been conducted among clinical-year medical students and interns in the Western Region of Saudi Arabia as a whole, including Makkah, Madinah, Jeddah, and Taif. Hence, the primary aim of our study is to evaluate the knowledge and attitudes regarding DNR decisions among clinical-year medical students and interns in the Western Region of Saudi Arabia.

## Materials and methods

Study design and participants

This cross-sectional study investigated the knowledge and attitudes of clinical-year medical students and interns regarding DNR decisions in the Western Region of Saudi Arabia. The participants were selected by convenience sampling. The inclusion criteria included medical students in clinical years and interns currently living in the Western Region of Saudi Arabia, aged 18 and older. Participants who declined to participate in the study or did not meet eligibility standards were excluded.

Ethical considerations and sample size

After obtaining institutional review board approval from Umm Al-Qura University’s Biomedical Ethics Committee (Approval No. HAPO-02-K-012-2023-10-1794), the study was carried out between October 2023 and December 2023. The data were acquired via an online self-administered questionnaire created using Google Forms and sent to clinical years medical students and interns. The sample size was estimated using the OpenEpi website, keeping the confidence interval at 95% [10]. The minimum sample size was calculated to be 385 participants.

Study tool and scoring

A validated questionnaire was obtained from a previously published study [11]. The questionnaire is divided into two parts: the first part contains demographic data of the targeted population, and the second part consists of 13 questions to assess the knowledge and attitudes toward DNR decisions among clinical-year medical students and interns in the Western Region of Saudi Arabia. 

Statistical analysis

The data analysis was carried out using IBM SPSS Statistics for Windows, Version 21 (Released 2012; IBM Corp., Armonk, New York, United States). All statistical methods used were two-tailed with an alpha level of 0.05 considering significance if the p-value is less than or equal to 0.05. Descriptive analysis was done by frequency distribution and percentage for study variables, including participants' personal data and academic data. In addition to medical students' knowledge and awareness about DNR and also their attitude level. Sources of information about DNR and factors to be considered when making the DNR decision were graphed. Cross tabulation for showing factors associated with medical students' knowledge and attitude about DNR was done using the Pearson Chi-square test and exact probability test for small frequency distributions. 

## Results

A total of 397 eligible participants were involved in the current study. Participants’ ages ranged from 20 to 29 years, with a mean age of 23.0 ± 1.4 years. Of the respondents, 258 (65%) were male. As for the academic year, 152 (38.3%) were in the fifth year, 73 (18.4%) were in the sixth year, and 102 (25.7%) were medical interns (Table 1).

**Table 1:** Socio-demographic data of the study participants (n = 397) SD: Standard deviation

Variable	No.	%
University		
Umm Al-Qura university	251	63.2%
Taibah University	79	19.9%
King Saud Bin Abdulaziz University for Health Sciences	29	7.3%
King Abdulaziz University	17	4.3%
Taif university	14	3.5%
Ibn Sina National College	6	1.5%
Fakeeh College for Medical Sciences.	1	.3%
Age in years		
20-21	49	12.3%
22-23	215	54.2%
24+	133	33.5%
Mean ± SD	23.0 ± 1.4	
Gender		
Male	258	65.0%
Female	139	35.0%
Academic year		
4th year	70	17.6%
5th year	152	38.3%
6th year	73	18.4%
Intern	102	25.7%

Table 2 shows that 364 (91.7%) of the respondents had heard about the DNR term, and 200 (50.4%) thought physicians should be involved in DNR decision-making. Conversely, 156 (39.3%) thought that this should be the patient. When the participants were asked, “Who has the right to know about a DNR decision?” 351 (88.4%) reported for parents, 245 (61.7%) for siblings, 245 (61.7%) for spouses, and 17 (5.3%) for others. Of the studied population, 226 (56.9%) identified the presence of a clear DNR policy in Saudi Arabia. In addition, 173 (43.6%) knew about the fatwa regarding DNR in Saudi Arabia. Furthermore, 77 (19.4%) of the participants indicated that they had prior experience with DNR. Of them, the experience involved first-degree relatives (42.9%), a patient (24.7%), a spouse (13%), and an uncle (6.5%). Additionally, more than three-quarters of the participants (333, 83.9%) were willing to take a lecture/session about DNR.

**Table 2:** Participants' knowledge and awareness about do-not-resuscitate decisions (n = 397) DNR: Do-not-resuscitate

Question	No.	%
Have you ever heard about the Do-Not-Resuscitate term?		
Yes	364	91.7%
No	33	8.3%
Who do you think should be involved in DNR decision-making?		
Physician	200	50.4%
Patient	156	39.3%
First-degree relatives	41	10.3%
Who has the right to know about DNR decision?		
Parents	351	88.4%
Siblings	245	61.7%
Spouse	245	61.7%
Patient	12	3.0%
Others	9	2.3%
Do you know if there is a clear policy regarding DNR in Saudi Arabia?		
Yes	226	56.9%
No	15	3.8%
I don't know	156	39.3%
Do you know if there is a fatwa regarding DNR in Saudi Arabia?		
Yes	173	43.6%
No	17	4.3%
I don't know	207	52.1%
Did you have a previous experience with DNR?		
Yes	77	19.4%
No	320	80.6%
If yes, with whom?		
First degree relatives.	33	42.9%
Patient	19	24.7%
Spouse.	10	13.0%
Uncle.	5	6.5%
A patient	4	5.2%
Friends.	3	3.9%
Others	3	3.9%
Are you willing to take a lecture/session regarding DNR?		
Yes	333	83.9%
No	64	16.1%

**Figure 1:**
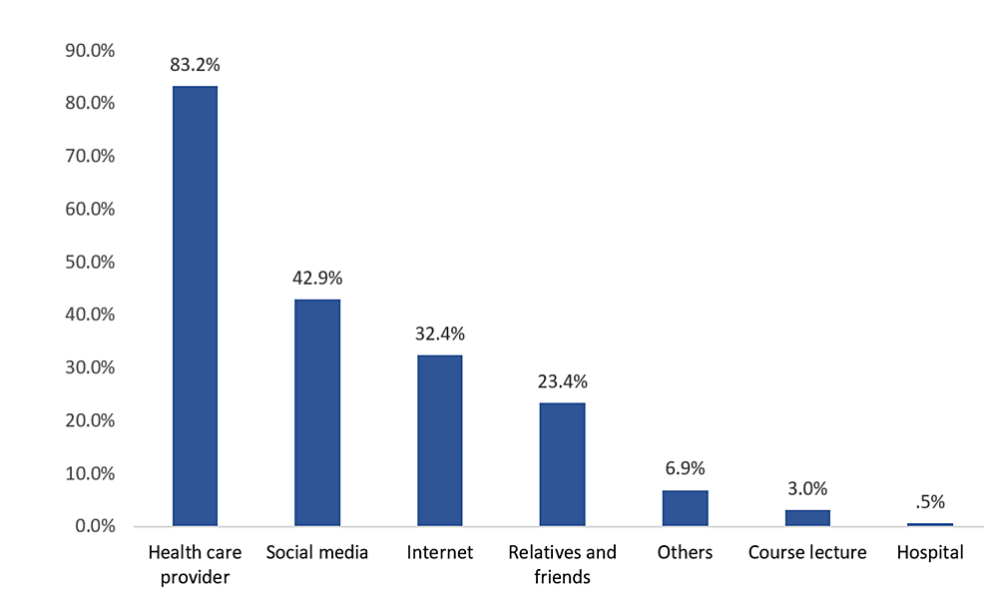
Sources of information about do-not-resuscitate decisions (n = 397)

The most common sources of information about DNR were healthcare providers (83.2%), social media platforms (42.9%), and the Internet (32.4%) (Figure 1).

**Figure 2:**
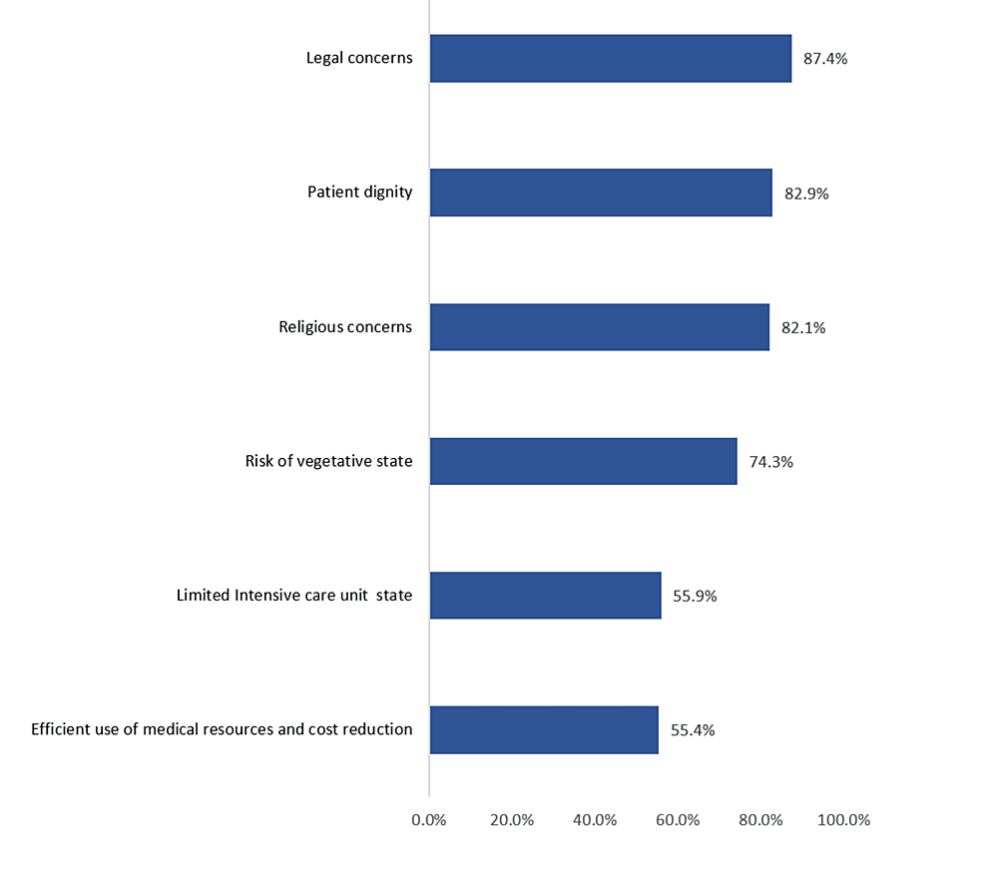
Factors reported to be considered when making do-not-resuscitate decisions (n = 397)

Figure 2 demonstrates the participants’ view on the factors to be considered when making a DNR decision. Of the participants, 347 (87.4%) and 326 (82.1%) reported legal and religious concerns, respectively. In addition, 329 (82.9%) opted for patient dignity, and 295 (74.3%) opted for the risk of a vegetative state. At the same time, 222 (55.9%) reported limited intensive care unit space and 220 (55.4%) for efficient use of medical resources and cost reduction.

A total of 55.2% of the participants had a positive attitude about DNR, and 72.5% agreed that the patient has the right to reject the DNR decision. Additionally, 38.5% of the participants agreed that it is acceptable to be conservative in investigations and treatments with DNR patients. In contrast, 47.9% found it unacceptable to withdraw life-sustaining treatment from DNR patients. Furthermore, 56.2% of the respondents disagreed with patients being kept unaware of their DNR status, while 51.6% agreed with encouraging organ donation discussions with DNR patients and/or their families. Moreover, 73% of the respondents felt that discussion about DNR order is stressful (Table 3).

**Table 3:** Participants' attitude toward do-not-resuscitate decisions (n = 397) DNR: Do-not-resuscitate

Statement	Agree		Neutral		Disagree	
	No.	%	No.	%	No.	%
Attitude toward DNR	219	55.2%	144	36.3%	34	8.6%
The patient has the right to reject DNR decision	288	72.5%	75	18.9%	34	8.6%
It is acceptable to be conservative in investigations and treatments with patients who are labeled as DNR patients.	153	38.5%	136	34.3%	108	27.2%
It is acceptable to withdraw life-sustaining treatment from DNR-labeled patients.	76	19.1%	131	33.0%	190	47.9%
The discussion of organ donation with DNR patients and/or their families should be encouraged.	205	51.6%	157	39.5%	35	8.8%
It is best that patients are not made aware of their DNR status.	64	16.1%	110	27.7%	223	56.2%
Discussion about DNR order is stressful.	290	73.0%	91	22.9%	16	4.0%

The association between the knowledge and attitudes about DNR decisions and the participants’ demographic characteristics is shown in Table 4 and Table 5. Interns demonstrated a greater degree of familiarity with the term DNR and a positive attitude regarding the DNR definition in comparison to fourth-year medical students (p ≤ 0.05). Moreover, participants who had heard about DNR and those who had previous experience with DNR conveyed a positive attitude compared to those who had not (p ≤ 0.05).

**Table 4:** Factors associated with participants' knowledge about do-not-resuscitate decisions DNR: Do-not-resuscitate P: Pearson X2 test; ^: Exact probability test; * p < 0.05 (significant)

Factors	Heard about Do-Not-Resuscitate				p-value
	Yes		No		
	No.	%	No.	%	
University					0.594^
Fakeeh College for Medical Sciences	1	100.0%	0	0.0%	
Ibn Sina National College	6	100.0%	0	0.0%	
King Abdulaziz University	16	94.1%	1	5.9%	
King Saud Bin Abdulaziz University for Health Sciences	29	100.0%	0	0.0%	
Taibah University	73	92.4%	6	7.6%	
Taif university	12	85.7%	2	14.3%	
Umm Al-Qura university	227	90.4%	24	9.6%	
Age in years					0.252
20-21	42	85.7%	7	14.3%	
22-23	198	92.1%	17	7.9%	
24+	124	93.2%	9	6.8%	
Gender					0.034*
Male	231	89.5%	27	10.5%	
Female	133	95.7%	6	4.3%	
Academic year					0.001*
4th year	53	75.7%	17	24.3%	
5th year	142	93.4%	10	6.6%	
6th year	68	93.2%	5	6.8%	
Intern	101	99.0%	1	1.0%	
Did you have a previous experience with DNR?					0.783
Yes	70	90.9%	7	9.1%	
No	294	91.9%	26	8.1%	

**Table 5:** Factors associated with participants' attitude toward do-not-resuscitate decisions DNR: Do-not-resuscitate P: Pearson X2 test; ^: Exact probability test; * p < 0.05 (significant)

Factors	Attitude toward DNR						p-value
	Agree		Neutral		Disagree		
	No.	%	No.	%	No.	%	
University							0.052^
Fakeeh College for Medical Sciences.	0	0.0%	1	100.0%	0	0.0%	
Ibn Sina National College	5	83.3%	1	16.7%	0	0.0%	
King Abdulaziz University	9	52.9%	7	41.2%	1	5.9%	
King Saud Bin Abdulaziz University for Health Sciences	25	86.2%	4	13.8%	0	0.0%	
Taibah University	41	51.9%	28	35.4%	10	12.7%	
Taif university	10	71.4%	4	28.6%	0	0.0%	
Umm Al-Qura university	129	51.4%	99	39.4%	23	9.2%	
Age in years							0.188
20-21	28	57.1%	17	34.7%	4	8.2%	
22-23	108	50.2%	84	39.1%	23	10.7%	
24+	83	62.4%	43	32.3%	7	5.3%	
Gender							0.011*
Male	136	52.7%	92	35.7%	30	11.6%	
Female	83	59.7%	52	37.4%	4	2.9%	
Academic year							0.001*
4th year	33	47.1%	25	35.7%	12	17.1%	
5th year	73	48.0%	63	41.4%	16	10.5%	
6th year	39	53.4%	30	41.1%	4	5.5%	
Intern	74	72.5%	26	25.5%	2	2.0%	
Have you ever heard about Do-Not-Resuscitate term?							0.001*
Yes	211	58.0%	127	34.9%	26	7.1%	
No	8	24.2%	17	51.5%	8	24.2%	
Did you have a previous experience with DNR?							0.001*
Yes	57	74.0%	19	24.7%	1	1.3%	
No	162	50.6%	125	39.1%	33	10.3%	

## Discussion

A DNR order refers to a clinical directive to refrain from performing CPR in the event of cardiac or pulmonary arrest while maintaining standard care and therapy. The knowledge and attitudes of medical students and interns regarding DNR decisions are crucial elements in critical care medicine. Hence, this study conducted a thorough investigation to evaluate the knowledge and attitudes regarding DNR decisions among clinical-year medical students and interns in the Western Region of Saudi Arabia.

Among the surveyed participants, our results showed that the vast majority of participants (91.7%) had heard about the term DNR. These results are consistent with what was found in the study by Abbas et al. (82.8%) [7]. It is notable that the interns exhibited more familiarity and positive attitudes toward the DNR term and definition compared to the fourth-year medical students (p ≤ 0.05), which can be attributed to variations in the level of expertise, knowledge, and clinical exposure. A comprehensive understanding of DNR orders is regarded as a valuable influencing factor when confronted with DNR decisions [8].

Regarding the source of information, most of the participants had heard the term DNR from healthcare providers (83.2%), which is higher than the study by Abbas et al. (60.51%) [7]. The concept of DNR may have been initially introduced to medical students via lectures or medical studies; thus, healthcare providers likely constituted their primary source.

Most of the respondents (50.4%) believed that physicians ought to be involved in DNR decision-making, which is correct based on the national DNR policy in Saudi Arabia [6], as more than half of the participants (56.6%) recognized the presence of a clear policy regarding DNR in Saudi Arabia, which might be due to the evolution of the medical curriculum and the integration of critical care subjects. In contrast, a significant proportion of the participants (49.6%) in the study by Abbas et al. selected the patient as the individual who ought to be involved and included in the DNR decision-making process, and 66.1% were not aware of the presence of the DNR policy [7]. Of the participants, 19.4% had a previous experience with DNR, and only 1.3% disagreed with the DNR definition, in contrast to 10.3% of the respondents with no previous experience (80.6%) (p ≤ 0.05). This raises a valid debate that medical students should be exposed to the intensive care unit (ICU) as a mandatory rotation during their clinical years.

Diverse factors influence the decision of a DNR order, as indicated by most participants in our study: legal and religious concerns, patient dignity, and risk of vegetative state are essential factors to consider when making a DNR decision; moreover, participants considered ICU space and cost reduction to be less important factors. These findings are consistent with several local studies [7,11,12]. Nevertheless, an international study at the University of Hong Kong observed that a patient’s age is a significant determinant in DNR decisions [8]. Furthermore, a study at the University of Tehran considered the leading factors to be the patient’s condition, religious concerns, the availability of ICU beds, and concerns regarding the patient’s and family’s reactions [9].

Most of the participants (72.5%) agreed that the patient has the right to reject a DNR decision. Additionally, the majority (56.2%) disagreed with patients being kept unaware of their DNR status. This demonstrates that medical students and interns place greater emphasis on patient autonomy.

DNR order discussion is stressful, as per our respondents (73%); this is essentially due to the sensitive and challenging nature of such a discussion. An additional study conducted in the Western Region of Saudi Arabia in 2015 among residents corroborates our results, as residents felt uncomfortable discussing DNR status with patients or their surrogates [13]. This emphasizes the gap in the education of end-of-life care skills, specifically regarding DNR, and highlights the significance of providing medical students, interns, and even residents with lectures and simulated training about DNR and end-of-life care-related skills, as more than three-quarters of our participants (83.9%) expressed their willingness to attend lectures or sessions regarding DNR.

Limitations

The present study investigated only a single region in Saudi Arabia. Hence, this might impact the generalizability of the results to other regions of Saudi Arabia. Additionally, sampling bias may constitute a possible limitation of this study. Given that the survey elicited a higher response rate from male participants than their female counterparts, a balanced sample could enhance the generalizability of the findings.

## Conclusions

Our current study assesses and evaluates the knowledge and attitudes regarding DNR decisions among clinical years medical students and interns in the Western Region of Saudi Arabia. Based on the gap that we found between clinical years medical students and interns, we think it is crucial to place a mandatory rotation in the intensive care unit, in addition to introducing lectures and simulated training about DNR and end-of-life care-related skills to prepare future physicians with knowledge and confidence when discussing DNR decisions.
